# Overexpression of RKIP and its cross-talk with several regulatory gene products in multiple myeloma

**DOI:** 10.1186/s13046-017-0535-z

**Published:** 2017-05-05

**Authors:** Anna Shvartsur, Kevin B. Givechian, Hermes Garban, Benjamin Bonavida

**Affiliations:** 1 Department of Microbiology, Immunology, and Molecular Genetics, David Geffen School of Medicine, University of California Los Angeles, Los Angeles, CA 90095 USA; 2 Department of Biological Sciences, USC Dana and David Dornsife College of Letters, Arts and Sciences at the University of Southern California, Los Angeles, CA 90089 USA; 3 California NanoSystems Institute (CnSI), University of California Los Angeles, Los Angeles, CA 90095 USA

**Keywords:** Cancer, Multiple myeloma, Prognosis, RKIP, Signaling cross-talks, Signature, Therapy

## Abstract

Multiple myeloma (MM) is a clonal plasma-cell neoplastic disorder arising from an indolent premalignant disease known as monoclonal gammopathy of undetermined significance (MGUS). MM is a biologically complex heterogeneous disease reflected by its variable clinical responses of patients receiving the same treatment. Therefore, a molecular identification of stage-specific biomarkers will support a more individualized precise diagnostic/prognostic approach, an effective therapeutic regime, and will assist in the identification of novel therapeutic molecular targets. The metastatic suppressor/anti-resistance factor Raf-1 kinase inhibitor protein (RKIP) is poorly expressed in the majority of cancers and is often almost absent in metastatic tumors. RKIP inhibits the Raf/MEK/ERK1/2 and the NF-κB pathways. Whereby all tumors examined exhibited low levels of RKIP, in contrast, our recent findings demonstrated that RKIP is overexpressed primarily in its inactive phosphorylated form in MM cell lines and patient-derived tumor tissues. The underlying mechanism of RKIP overexpression in MM, in contrast to other tumors, is not known. We examined transcriptomic datasets on Oncomine platform (Life Technologies) for the co-expression of RKIP and other gene products in both pre-MM and MM. The transcription of several gene products was found to be either commonly overexpressed (i.e., RKIP, Bcl-2, and DR5) or underexpressed (i.e., Bcl-6 and TNFR2) in both pre-MM and MM. Noteworthy, a significant inverse correlation of differentially expressed pro-apoptotic genes was observed in pre-MM: overexpression of *Fas* and *TNF-*α and underexpression of *YY1* versus expression of anti-apoptotic genes in MM: overexpression of *YY1* and underexpression of *Fas* and *TNF-*α. Based on the analysis on mRNA levels and reported studies on protein levels of the above various genes, we have constructed various schemes that illustrate the possible cross-talks between RKIP (active/inactive) and the identified gene products that underlie the mechanism of RKIP overexpression in MM. Clearly, such cross-talks would need to be experimentally validated in both MM cell lines and patient-derived tumor tissues. If validated, the differential molecular signatures between pre-MM and MM might lead to a more precise diagnosis/prognosis of the disease and disease stages and will also identify novel molecular therapeutic targets for pre-MM and MM.

**Electronic supplementary material:**

The online version of this article (doi:10.1186/s13046-017-0535-z) contains supplementary material, which is available to authorized users.

## Background

Multiple Myeloma (MM) is an incurable hematological malignancy of plasma cells with low proliferation in the bone marrow. Patients with MM account for 10% of deaths caused by blood cancers and have a median survival of approximately 5 years. Uncontrolled growth can lead to bone marrow failure, which can cause anemia, thrombocytopenia and leukopenia. In addition, MM has been associated with renal dysfunction. MM cells interact with nonmalignant cells in the bone marrow microenvironment, enhancing bone loss and osteoporosis, causing severe pain. Together, MM biology is complex and warrants a deeper molecular understanding to subsequently enhance MM therapy [1, 2].

Several mechanisms have been proposed underlying both the innate and acquired resistance of MM to various therapies, which include the activation of survival/anti-apoptotic pathways, such as the PI3K/AKT, JAK/STAT3, TGFβ/β-catenin, Wnt, Notch, IGF, and NF-κB pathways [3–10]. In agreement, we have previously reported a dysregulated NF-κB/YY1/Snail/RKIP/PTEN loop detected in many cancers and showed that it promotes cell survival, proliferation, induces an epithelial to mesenchymal transition (EMT), potentiates invasion and metastasis, maintains drug/immune resistance, promotes anti-apoptotic mechanisms, and regulates the cancer stem cell phenotype [11, 12]. In short, high RKIP expression inhibits NF-κB, thereby inhibiting Snail, YY1, AKT while inducing PTEN and pro-apoptotic factors. Conversely, with low RKIP expression, the opposite holds true [13–15]. Noteworthy, unlike most tumor cells, we have previously reported that MM cells overexpress the inactive phosphorylated form of RKIP and, therefore, is of consideration in our present study [11]. While several comprehensive reviews of RKIP have recently been reported [16–20], we specifically investigated the expression of RKIP/p-RKIP and potential cross-talk pathways of correlated gene products in both multiple myeloma (MM) and pre-MM, using a subset of relevant genes in the publicly available Oncomine database.

## Differential RKIP expression and activity in cancer and MM

The human *RKIP* gene is located on chromosome 12q24 and contains a promoter region with several sequences involved in the regulation of RKIP transcription, such as the E1-Box, E2-Box, CpG islands, and androgen-response elements (ARE) [21–23]. Like many proteins, RKIP can undergo post-translational phosphorylation modifications, which alter its conformation-dependent function. Specifically, mediated by its isoenzymes (α,-βI, −βII, −γ, and atypical ζ) and in response to growth factor stimuli, PKC phosphorylates RKIP at Ser153, thus inducing a structural, inactivating change in the RKIP ligand-binding pocket, causing RKIP to dissociate with Raf-1 and thereby activating the ERK/MAPK pathways [24–27]. On the other hand, unphosphorylated RKIP may bind MEK and ERK, causing Raf-1-MEK dissociation and ERK signaling mitigation. When observing RKIP activity during the cell cycle of cultured mammalian cells, hyperactivation of the MAPK pathway in RKIP-deficient cells displayed decreased localization of phosphorylated and active Aurora B to kinetochores, thus indicating RKIP activity in spindle checkpoint regulation [28, 29]. Indeed, in an RKIP-dependent manner during G1/S and G2/M transitions, RKIP influenced cell cycle kinetics as its overexpression was found to reduce cell growth and proliferation rate, therefore suggesting a tumor suppressive role for the endogenous kinase inhibitor [30].

RKIP was reported to inhibit the expressions of both the Raf/MEK/ERK and the NF-κB signaling pathways through a physical interaction with B-Raf and an auto-regulatory feedback loop, respectively [15, 31–33]. RKIP is often referred to as PEPB1 (phosphatidyl ethanolamine binding protein 1) as member of the PEBP family of proteins and is expressed in almost all normal tissues in mammals [34]. However, RKIP expression is reduced in many cancers and is known to be a tumor metastasis suppressor gene product, explaining its downregulation during metastasis [34–40]. RKIP modulates apoptotic pathways, and its overexpression has been shown to reverse tumor chemo- and immune-resistance [41, 42]. Furthermore, a dysregulated NF-κB/Snail/YY1/RKIP loop can cause tumor cell resistance to cytotoxic drugs via inhibition of apoptosis [41, 42].

RKIP also regulates glycogen synthase kinase 3 (GSK3B) by both binding and maintaining GSK3β protein levels as well as inhibiting the activation of GSK3β targets. Therefore, RKIP indirectly inhibits the cell cycle inducing gene cyclin-D1 as well as the EMT and invasion inducing genes *β-catenin*, *Snail* and *Slug* [43]. RKIP also maintains a subdued oxidative stress response in cells in an unclear mechanism [30].

## Regulation of RKIP expression and its biologic functions

Snail is a transcriptional repressor of RKIP that binds to E-box *cis*-elements in the RKIP promoter and recruits mSin3A histone deacetylases and transcriptional repressor complexes [44, 45]. In the presence of Snail, the enhancer of Zeste homolog 2 (EZH2) inhibits RKIP expression at the transcriptional level, which hastens cellular invasion. RKIP gene expression can also be inhibited by microRNAs as the RKIP 3’-UTR contains a target identified by miR-224 in breast cancer cell lines [46]. Conversely, the overexpression of miR-let-7, miR-1, and miR-16 have been shown to mildly enhance RKIP protein translation, whereas overexpression of miR-155 has a destabilizing effect on RKIP protein stability [45, 47, 48].

NF-κB positively regulates both Snail and Yin Yang 1 (YY1), which further inhibit RKIP, which has similarly been shown to inhibit Snail transcription itself [44, 45, 49]. Indeed, upregulated RKIP was observed to act in a feedback mechanism by which NF-κB, Snail, and YY1 were inhibited.

As the only known physiological negative regulator of the MAPK pathway, RKIP is also a key player in cellular migration through its negative regulation of NF-κB and its target pro-invasion MMPs [30, 50–53]. Interestingly, RKIP induction additionally inhibits the EMT-related genes *MAPK*, *Myc*, *lin28*, *let7*, *vimentin* and *fibronectin*, while upregulating the epithelial genes *E-cadherin* and *cytokeratin* 18 [45, 49]. Its overexpression can also induce tumor cell sensitivity to TRAIL apoptosis, perhaps due to consequential inhibition of YY1 and upregulation of DR5 [54, 55]. Immunologically, active *RKIP* may serve as a cancer surveillance gene, since its low expression in tumor cells allows their immune cytotoxic effector cell evasion [55].

## RKIP Expression in multiple myeloma

Low RKIP expression is found in many cancers, such as bladder, breast, gastric, non-small cell lung, prostate, and other cancers [20, 56–58]. In metastatic melanoma, downregulation of both RKIP and phosphorylated RKIP has been noted, while low RKIP and high-phosphorylated RKIP expression may be indicative of non-metastatic melanoma [59].

For most solid tumors, the majority of studies has shown that loss of RKIP may be of prognostic significance with regard to overall survival, disease free survival, and presence of metastasis [60]. In MM, RKIP expression is high. RKIP and the inactivated p-Ser153 form of RKIP are overexpressed in multiple myeloma cell lines and patients’ tissues compared to other tumors, healthy B cells, and healthy bone marrow. Specifically, about half of the RKIP positive cells in MM are in the phosphorylated form. This high RKIP expression in MM is positively correlated with a more aggressive diagnosis usually resulting in a worse prognosis. It also correlates with other aggressive prognostic factors, such as IgH translocations, which have been shown to increase mortality. Thus, it is possible that the high expression of inactive RKIP in MM may allow for the constitutive activation of survival pathways and cell growth and proliferation [11]. The relationship between the expression of RKIP in MM and the regulatory gene products in MM was analyzed below.

### Correlation Between RKIP Expression in MM and Various Gene Products Expressed in MM: Criteria for Selection

Bioinformatics analyses were performed via information found in the Oncomine Database at https://www.oncomine.org. A differential screen was selected for cancer vs. normal tissue, MM being the selected cancer, and mRNA being the parameter used for the datasets. The following gene products were analyzed for their relative associations with the reported activation of RKIP: AKT, Bcl-2, Bcl-6, CIAP1, DR5, E-cadherin, Fas, FasL, NF-κB, PTEN, SNAI1, SNAI2, TNF-α, TNFR-1, TNFR-2, TRAIL, XIAP, and YY1 [11, 13, 14, 41, 54, 61–65]. These gene products were selected because of their determined or hypothesized importance in multiple myeloma based on the reported literature. Three datasets were initially analyzed, namely, the Agnelli Myeloma 3 (Dataset 1), the Zhan Myeloma (Dataset 2), and the Zhan Myeloma 3 (Dataset 3). Each dataset used bone marrow plasma cell tissues for their analyses. Dataset 1 has 133 MM and 5 plasma cell leukemia (PLK) samples with a *p*-value set to ≤ 0.05 [66]. Dataset 2 has 74 MM, 37 plasma cell, 7 tonsillar lymphomas and 31 healthy samples with a *p*-value set to ≤ 0.0001 [67]. Dataset 3 lacked of MM samples, containing only bone marrow and smoldering myeloma samples and, thus, was not included in the final analysis due to this small number of samples [68].

**Fig. 1:**
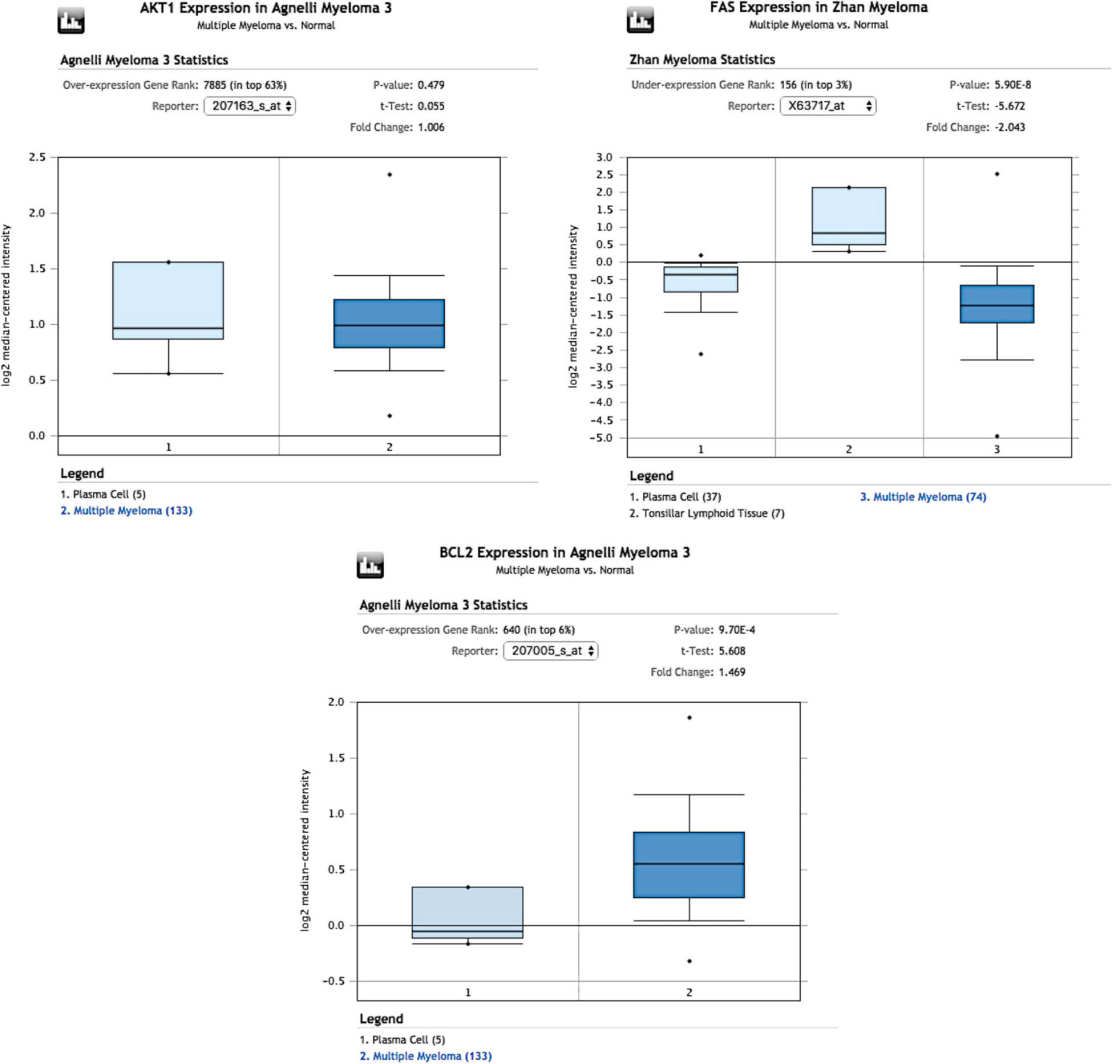
Oncomine log2-mediated intensity graphs for three examples. The graphs represented were derived from the Oncomine database and indicate the expression profiles of three different gene product in MM: **a** No significant overexpression for AKT; **b** Significant underexpression of Fas; **c** Significant overexpression of Bcl-2

Figure 1 provides an example of the Oncomine log2-mediated intensity graphs for three gene products (AKT, Fas, and Bcl-2) that exhibited different expression states: no significant expression for AKT (Fig. 1a), significant underexpression for Fas (Fig. 1b), and significant overexpression for Bcl-2 (Fig. 1c). For dataset 1 and for dataset 2, p-values of ≤ 0.05 and ≤ 0.0001, respectively, were considered as significant according to the respective publications [66, 67]. The analysis of the various gene products and their correlations with RKIP were examined using the same methods used in Fig. 1.

**Fig. 2:**
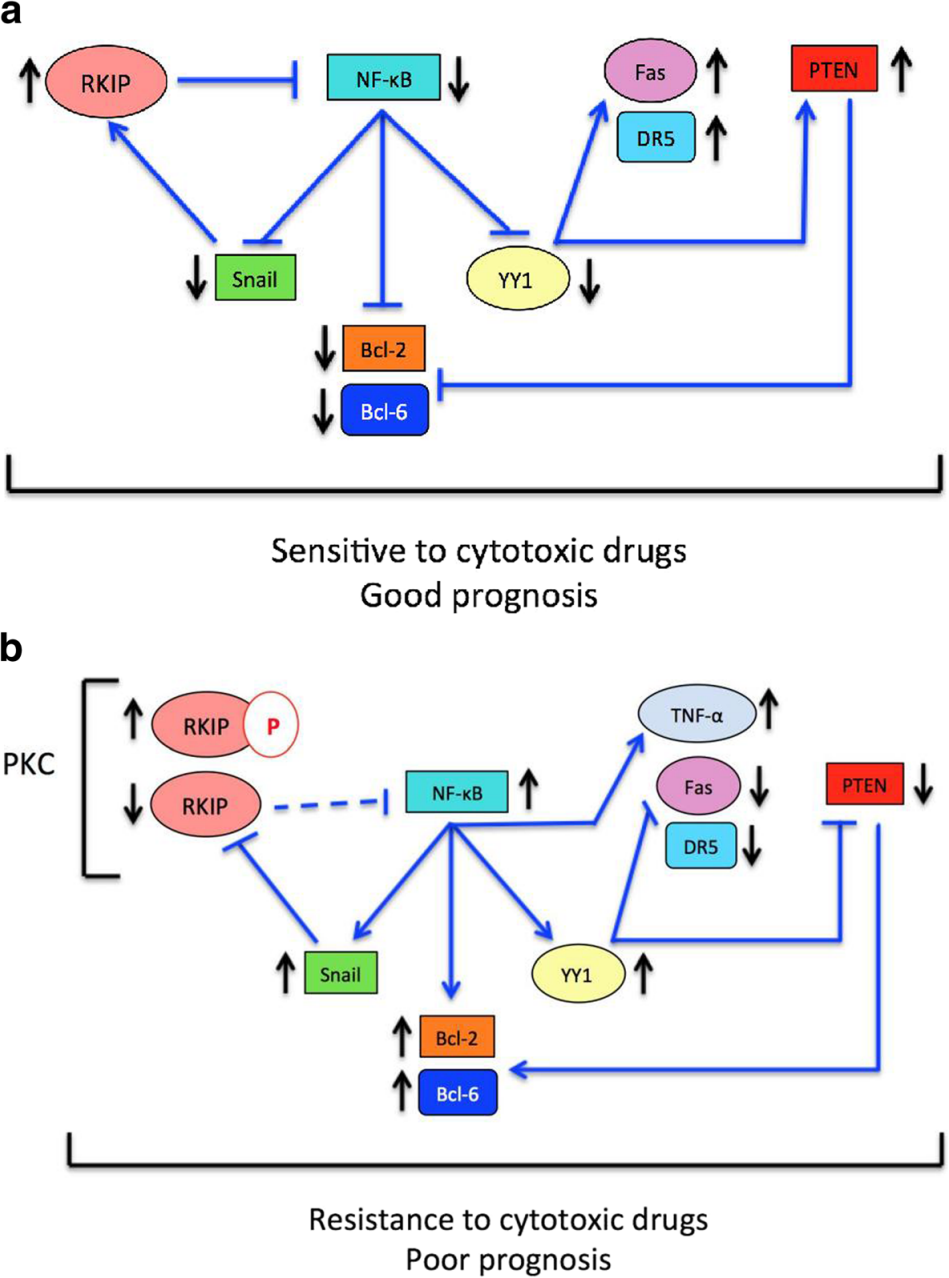
**a** MM Expressing Active RKIP. *Blue arrows* signify the interactions of gene products. *Black arrows* signify whether the expression of the associated gene product is increased or decreased. If RKIP expression is assumed to be high and functional, then high DR5 expression would be expected through the action of NF-κB inhibiting YY1 [14]. PTEN is expected to be highly expressed through the action of NF-κB inhibiting YY1, which will no longer inhibit PTEN, thus causing PTEN overexpression [13]. Bcl-2 is overexpressed in the data, but according to findings in literature, if RKIP is overexpressed, Bcl-2 should be inhibited via NF-κB and PTEN [61, 69]. In the outlined pathway, Fas is expected to be overexpressed, but in the data, Fas is overexpressed in MM [13]. No clear relationship is determined with RKIP overexpression and Bcl-6, TNFR-2, TRAIL, and TNF-α. **b** MM Expressing Inactive, Phosphorylated RKIP. Blue arrows signify the interactions of gene products. *Black arrows* signify whether the expression of the associated gene product is increased or decreased. In multiple myeloma, high RKIP expression often corresponds to phosphorylated RKIP, which is inactive [11]. Inactive (“low” or phosphorylated RKIP) would correspond to TNF-α overexpression via the expression of NF-κB, since inactive RKIP may not be able to inhibit NF-κB [70]. This would also correspond to underexpression of Fas via NF-κB's activation of YY1, which would inhibit Fas [13]. The overexpression of Bcl-2 would be explained via the inactivation of PTEN via YY1 expression and the activation of Bcl-2 with NF-κB [61, 69] However, in the data, PTEN and DR5 are overexpressed and Bcl-6 is underexpressed, which is opposite to their expected expression found literature findings [13, 62]. There is no clear relationship between RKIP inactivation and TNFR-2 and TRAIL levels

Figure 2a, b represents the various pathways that lead to the expression of the various gene products under the conditions that the cancer cells express active RKIP (Fig. 2a) and inactive RKIP (Fig. 2b). These various pathways have been represented in the literature [11, 13, 14, 61, 62, 69, 70].

The Oncomine data were used to examine the mRNA levels of representative gene products in MM and whether or not they correlated with the expected findings summarized in Fig. 2. It must be noted that due to the heterogeneity of the MM cell population, it is likely that there exist several subsets expressing various forms of RKIP, namely a subset expressing exclusively RKIP, a subset expressing exclusively pRKIP, a subset expressing both RKIP and pRKIP, and a subset not expressing either form of RKIP. Since the Oncomine analysis and the represented literature analysis were performed with the unselected cell populations, the overall findings will vary according to the levels of RKIP and pRKIP and their proportions within the populations under analysis.

The following analyses of the Oncomine data of various gene products are shown in Table 1.

**Table 1:** Compilation of bioinformatics MM Gene product expression data

Gene Product	Overexpression	Underexpression
Bcl-2	2/2	0/2
TRAIL	2/2	0/2
DR5	1/1	0/1
RKIP	1/2	0/2
PTEN	1*/2	0/2
TNF-α	1*/2	0/2
Bcl-6	0/2	1/2
Fas	0/2	1/2
TNFR-2	0/2	1/2
AKT	0/2	0/2
CIAP1	0/2	0/2
E-cadherin	0/2	0/2
FasL	0/2	0/2
NF-κB	0/2	0/2
SNAI1	0/1	0/1
SNAI2	0/1	0/1
TNFR-1	0/2	0/2
XIAP	0/1	0/1
YY1	0/2	0/2

Numeral values in table represent absolute significant ratios of examined datasets

1*(Agnellie Myeloma 3): ≤ 0.05

1 (Zhan Myelona): ≤ 0.0001

1*(Agnellie Myeloma 3): 37 MM; 3 PLK (Agnelli et al. 2009) [66]

1 (Zhan Myelona): 74 MM; 5 MGUS (Zhan et al. 2002) [67]

#### RKIP

RKIP is downregulated in most cancers, but in MM, there is high RKIP expression, but it is in its phosphorylated (or inactive pRKIP form). Phosphorylated RKIP antagonizes unphosphorylated RKIP in inhibiting survival signals and promoting apoptosis. Phosphorylation represses the activity of RKIP as an antisurvival factor. In the bioinformatics data, RKIP was found to be overexpressed in MM, in agreement with our reported findings, although it did not discriminate between active RKIP or pRKIP [11].

#### AKT

In the bioinformatics data, AKT was not significantly over or underexpressed.

#### NF-κB

NF-κB is a protein complex that may act as an antiapoptotic factor in MM cells. NF-κB protects MM cells from apoptosis both by maintaining mitochondrial stability via Bcl-2 expression, as well as by attenuating death-receptor–induced apoptosis via CIAP1 and CIAP2 expression [71]. The ReIB subunit of NF-κB is found to induce the expression of anti-apoptotic genes *CIAP2*, *Bcl-XL*, and *Bcl-2* to promote cell survival [63]. The NF-κB pathway is both directly and indirectly implicated in regulating EMT through the transcription and expression of gene products that participate in the EMT cascade, such as Snail, the metastasis-inducer, and E-cadherin suppressor transcription factors. Snail represses RKIP which, when actively expressed, inhibits the Raf-1/MEK/ERk and NF-κB survival pathways implicated in EMT [13].

Low active RKIP would thus be expected to result in high NF-κB expression. However, no significant variation in expression of NF-κB was found in either of the two datasets of the bioinformatics data we investigated – specifically in regard to NF-κB1, the nuclear factor of kappa light polypeptide gene enhancer in B cells (Oncomine).

#### YY1

NF-κB is a protein complex that regulates the transcription of both Snail and YY1 (Ying Yang 1). YY1 positively regulates Snail and, thus, indirectly represses RKIP. Additionally, there are preliminary findings showing YY1 may directly repress RKIP by binding to its promoter. In either case, it is known that upregulated RKIP inhibits NF-κB activation via a well-documented feedback mechanism, and this, as expected, results in the inhibition of downstream NF-κB effectors, namely, Snail and YY1. However, if NF- κB is phosphorylated, the opposite holds true, at least in part, due to its inactivity [13].

Specifically, YY1 has been reported to be overexpressed in most MM tumor cells [72], which leads to the expectation that expression of YY1 and pRKIP would be upregulated in MM [54]. Despite this expectation, the bioinformatics MM data did not show significant overexpression. Interestingly, however, in pre-MM, YY1 was significantly underexpressed.

#### SNAI1

SNAI1, often referred to as Snail, can transcriptionally inhibit RKIP [44]. Likewise, RKIP has been shown to inhibit the transcription of Snail at the mRNA level [45]. Snail would be expected to be upregulated in MM, considering pRKIP does not interfere with the dysregulated NF-κB/YY1/Snail/RKIP/PTEN loop [14]. In the bioinformatics MM data analyzed, Snail was neither significantly overexpressed nor underexpressed in agreement with previous studies [73].

#### SNAI2

In the bioinformatics data analyzed, SNAI2, also known as slug, was not significantly over or underexpressed.

#### PTEN

PTEN (phosphatase and tensin homolog), which is inhibited by both YY1 and Snail (which are regulated by NF-κB), would be expected to be underexpressed with a dysregulated RKIP loop and low RKIP or high pRKIP [14]. However, in the bioinformatics data, PTEN is actually overexpressed in MM in agreement with high levels of RKIP in a subset of MM cells.

#### Bcl-2

The Bcl-2 (B-cell lymphoma 2) family of proteins plays a pivotal role in regulating Cyt C release and apoptosis. Bcl-2 and Bcl-XL are anti-apoptotic molecules, while, Bax and Bid are pro-apoptotic. Bcl-2 can block the release of Cyt C from the mitochondria and prevents the activation of caspase 3, while Bax and Bid can promote Cyt C release from the mitochondria and activate the caspases. Most death modulators act via Bcl-2-family proteins to regulate Cyt C release [74]. Bcl-2 is cleaved downstream of caspase activation, which shows a potential feedback amplification of caspase-induced mitochondrial dysfunction in IFN-induced apoptosis [74]. Type I but not type II interferons (IFNs) induce typical apoptosis through up-regulation of the TRAIL pathway and modulation of the Bcl-2 family of proteins in MM [74]. The mitochondria-dependent pathway can be inhibited by Bcl-2 [75]. Bcl-2 is a target of NF-κB. NF-κB activation induces the expression of pro-survival Bcl-2 proteins [61]. Thus, low active RKIP should result in high NF-κB and high Bcl-2 expression. Bcl-2 has been reported to be upregulated in MM cells and leads to chemoresistance for many drugs [76]. In agreement, the bioinformatics data show significant Bcl-2 overexpression for both MM and Pre-MM.

#### Bcl-6

Bcl-6 (B-cell lymphoma 6) is upregulated in the bone marrow microenvironment in multiple myeloma cells [62]. NF-κB mediates TNF-α- induced Bcl-6 expression. Bcl-6 expression is modulated, at least in part, via Janus kinase/STAT3 and NF-κB pathways [62]. Thus, low active RKIP or high pRKIP should be expected to result in decreased Bcl-6 expression due to the mediation of TNF-α- induced Bcl-6 expression by NF-κB [62]. Activation of CD40 receptors in B cell lymphoma leads to NF-κB- mediated IRF4 transcription factor induction, which represses Bcl-6 expression by binding to the Bcl-6 promoter [77]. In the bioinformatics data, Bcl-6 was found to be underexpressed in MM, but overexpressed in Pre-MM.

#### XIAP

RKIP overexpression contributes to the downregulation of anti-apoptotic gene products, such as XIAP (X-linked inhibitor of apoptosis protein). Specifically, XIAP is a target of NF-κB [54]. A dysregulated RKIP would result in NF-κB expression and, thus, XIAP expression. MM cell lines usually express high levels of XIAP [78]. In the bioinformatics data, XIAP had no significant variation.

#### CIAP1

RKIP overexpression contributes to the downregulation of CIAP1 (cellular inhibitor of apoptosis 1), an anti-apoptotic gene product [54]. The anti-apoptotic gene products CIAP1 and CIAP2 are recruited to the signaling complex of TNFR-1 via their interaction with both the TNF-receptor–associated factor 1 (TRAF1) and TRAF2, exerting an inhibitory effect on caspase-8 activation. There is a fundamental role for CIAP1/CIAP2 in regulating B-cell survival and responsiveness, which requires direct binding to TRAF2. Mutations of TRAF2, TRAF3, and CIAP1/CIAP2 can contribute to MM [79]. Deletion of CIAP1 plus CIAP2 (but not either alone) leads to uncontrolled accumulation of primary B cells in vivo [79]. It is also demonstrated that both CIAP1 and CIAP2 are necessary for TNF-α-mediated NF-κB activation [80]. Overexpression of CIAP1 and CIAP2 can cause cells to be highly resistant to TNF-α–induced apoptosis, by blocking the activation of caspase-8. CIAP1 is regulated by NF-κB, while CIAP1 and/or CIAP2 also mediate, at least in part, the protective effect of NF-κB against apoptosis in MM cells [71]. Thus, a dysregulated RKIP loop would result in NF-κB expression and thus CIAP1 expression. In the bioinformatics data, CIAP1 showed no significant variation in expression.

#### E-cadherin

Overexpression of RKIP results in the upregulation of epithelial gene products related to metastasis suppression, such as E-cadherin [13]. E-cadherin is inhibited by Snail expression [65]. Overexpression of Snail in cancer is partly responsible for inducing EMT through downregulation of E-cadherin [81]. Coupled positive feedback loops made up of ERK phosphorylating RKIP and Snail transcriptionally repressing RKIP have a role in causing a switch-like behavior of E-cadherin expression. RKIP expression inhibits EMT progression by preventing E-cadherin suppression [65]. Production of interleukin-17 (IL-17) in MM cells, leads to repression and, thus, low expression of E-cadherin [82]. Thus, in MM, low active RKIP would be expected to result in low E-cadherin. E-cadherin is negatively regulated by Snail and since Snail was not overexpressed (see SNAI1 and above SNAI2), it was expected that E-cadherin would not be overexpressed with high expression of pRKIP. The bioinformatics data shows no significant variation of the expression of E-cadherin.

#### TNF-α, TNFR-1, and TNFR-2

TNF-α is a survival factor for MM cell lines, although it is less potent than interleukin-6 (IL-6), which is a major survival factor for malignant plasma cells. TNF-α is produced in MM cells. TNF-α causes MM cell proliferation by inducing MM cells into the cell cycle and promotes long term malignant plasma cell line growth [83]. A large majority of MM cell lines express the TNF-α receptor 2, TNFR-2, while a large subset fails to show TNFR-1 expression [84]. The canonical (classical) pathway is partly activated through the TNF receptor family, while the apoptotic extrinsic pathway is mediated by TRAIL. TNFR-1 is the main pro-inflammatory TNF receptor. In T cells, TNFR-2, but not TNFR-1, is shown to induce activation of the non-canonical (alternative) pathway. Extrinsic apoptosis is induced by the interaction of the pro-apoptotic ligands: TRAIL, TNF-α or FasL to their specific receptors on infected or damaged cells [63]. A large majority of MM cell lines express TNFR-2, while a large subset fails to show TNFR-1 expression [84].

TNF-α is secreted into the bone marrow microenvironment by MM cells and induces NF-κB–dependent up-regulation of adhesion molecules on both MM cells and bone marrow stromal cells, resulting in increased adhesion. This binding confers resistance to apoptosis and triggers NF-κB–dependent secretion of IL-6. Constitutive activity of NF-κB in MM cells gets rid of the proapoptotic effect of TNF-α, due to the up-regulation of CIAP1 and CIAP2, and induces some proliferation. NF-κB regulates TNF-α. TNF-α can induce NF-κB nuclear translocation, CIAP1 and CIAP2 upregulation, and MM cell proliferation [75]. The TNF-α autocrine-paracrine loop can induce constitutive activation of NF-κB and YY1 in tumor cells [63]. Thus, low RKIP should result in the expression of TNF-α. In the bioinformatics data, TNF-α was overexpressed in both Pre-MM and MM. TNFR-2 was underexpressed in MM.

#### Fas and FasL

The system of Fas and Fas ligand (FasL) participates in the extrinsic apoptotic system. Their levels are increased in MM and are in parallel with disease stage and may thus reflect MM progression [85]. FasL plays an important role in eliminating target cells by effector T lymphocytes and in suppressing cell immune responses against malignant and nonmalignant cells. MM cells have been found to express Fas [86]. FasL has been shown to be constitutively expressed on the surface of MM cells in a study by Villunger et al. [87]. However, MM cells are highly resistant to Fas-mediated apoptosis [88].

Many tumors are resistant to FasL-induced apoptosis. This was tested in prostate cancer cells. The TNF autocrine-paracrine loop induces constitutive activation of transcription factors NF-κB and the transcription repressor YY1 in tumor cells, which both downregulate Fas and induce resistance to FasL-induced apoptosis [63]. Fas is transcriptionally regulated by interferons. Interferon-induced upregulation of Fas has been shown to sensitize MM cells to Fas-mediated apoptosis. An increased basal expression of Fas is associated with a higher sensitivity to Fas-mediated apoptosis [88]. Fas is repressed by YY1. Thus, a dysregulated RKIP loop should result in Fas downregulation [13]. In the MM bioinformatics data, Fas is underexpressed in agreement with the overexpression of pRKIP, while FasL was overexpressed, in agreement with the overexpression of RKIP. However, in the Pre-MM bioinformatics data, Fas is overexpressed.

#### TRAIL

TRAIL expression has been shown to be significantly higher in MM cells than plasma cells from MGUS (monoclonal gammopathy of undetermined significance) patients in a study by Kawano et al. [64]. It has been detected in the myeloma cell cytoplasm. TRAIL expression in MM cells has been correlated with the concentration of TRAIL in the bone marrow plasma. TRAIL expression is positively correlated with osteolytic markers, which suggests that the TRAIL produced from myeloma cells may have a role in bone resorption in MM patients [64].

TRAIL plays an important role in NK cell-mediated immuno- surveillance against tumors. TRAIL was shown to induce apoptosis in MM cells protected against MM cell-induced lytic bone destruction in vitro and in a mouse model [89]. TRAIL has been suggested to have a role in mediating interferon-α- induced apoptosis [88]. AP-1, a downstream transcription factors in the MAPK signaling cascade, acts as one of the key positive transcription factors for the expression of TRAIL. Upregulation of TRAIL receptors DR4 and DR5 expression is linked to the activation of the ERS (endoplasmic reticulum stress) response in various cancer cell lines. ERS has been found to induce AP-1 activation and TRAIL expression in macrophages. TRAIL expression in activated monocytes and macrophages may help prevent tumor growth [90].

The NF-κB pathway plays a key role in the regulation of TRAIL expression in activated human NK cells, indicating that the proteasome is involved in the regulation of TRAIL expression through the modulation of NF-κB activity. Proteasome inhibition may selectively impair NK cell cytotoxicity against myeloma cells and other TRAIL-sensitive tumor cells, possibly resulting in an impairment of immunosurveillance against such tumors. TRAIL expression on resting NK cells is undetectable or very low, but inducible by IL-2 stimulation (NK cells express very low level of TRAIL). IL-2 significantly induces TRAIL expression [89]. NF-κB inhibition can increase the sensitivity of MM cells to TRAIL [71]. Thus, overexpression of RKIP, which would inhibit NF-κB expression, would be expected to correlate with uninhibited TRAIL expression, whereas high pRKIP would be expected to result in low TRAIL expression. In the bioinformatics data, TRAIL was overexpressed in MM.

#### DR4 and DR5

RKIP overexpression was paralleled with the up-regulation of DR5 (TRAIL-R2), no change in DR4, and inhibition of YY1 in the Oncomine data. DR5 is a proapoptotic receptor, whose expression is mediated by NF-κB. NF-κB differentially regulates D5. NF-κB can directly bind to DR5 to activate its expression under certain conditions. For instance, the anti-apoptotic stimulus EGF can activate NF-κB and induces its p65 subunit to bind to the *DR5* gene, but fails to increase DR5 expression, while histone deacetylase 1 (HDAC) inhibitors can activate NF-κB and p53 to upregulate DR5 expression. Thus, cooperation with p53 and the NF-κB p65 subunit is important for DR5 expression [91]. MM cells have been found to express DR4 or DR5 [86]. High expression and variable expression of DR4 have been found in MM cell lines in a study by Mitsiades et al. [92]. DR5 is under the negative regulation of the transcription factor repressor YY1, a target of NF-κB [41]. RKIP-induced sensitization to TRAIL may be due to the inhibition of YY1 and upregulation of DR5. In the presence of inactive RKIP in MM, DR5 would be expected to be downregulated. RKIP overexpression may regulate tumor sensitivity to death ligands by inhibiting YY1 and up-regulating death receptors [41]. In the bioinformatics data, DR5 is overexpressed in MM in agreement with the overexpression of RKIP.

## Conclusions

After reviewing the Oncomine database, the gene rank and p-value information mined from Oncomine for each selected gene product with regard to overexpression and underexpression of the MM case and the Pre-MM (MGUS) case are shown in Additional file 1: Table S1. These data were then summarized to depict gene products that were overexpressed, underexpressed, or not significantly expressed in either case, and listed in Tables 1 and 2. Table 1 summarizes the data for the MM condition and Table 2 summarizes the data for the Pre-MM condition considering the published significance values. Gene products with significant overexpression in MM included Bcl-2, TRAIL, DR5, RKIP, PTEN, and TNF-α (Table 1). Gene products with significant underexpression in MM included Bcl-6, Fas, and TNFR-2 (Table 1). Gene products with significant overexpression in Pre-MM included Bcl-2, Bcl-6, FasL, and TNF-α (Table 2). YY1 was found to have significant underexpression in Pre-MM (Table 2).

**Table 2:** Compilation of Bioinformatics Pre- MM Gene product expression data

Gene Product	Overexpression	Underexpression
Bcl-2	1*/2	0/2
Bcl-6	1*/2	0/2
FasL	1*/2	0/2
TNF-α	1*/2	0/2
YY1	0/2	1*/2
AKT	0/2	0/2
CIAP1	0/2	0/2
DR5	0/1	0/1
E-cadherin	0/2	0/2
Fas	0/2	0/2
NF-κB	0/2	0/2
PTEN	0/2	0/2
RKIP	0/2	0/2
SNAI1	0/1	0/1
SNAI2	0/1	0/1
TNFR-1	0/2	0/2
TNFR-2	0/2	0/2
TRAIL	0/2	0/2
XIAP	0/1	0/1

Numeral values in table represent absolute significant ratios of examined datasets

1*(Agnellie Myeloma 3): ≤ 0.05

1 (Zhan Myelona): ≤ 0.0001

1*(Agnellie Myeloma 3): 37 MM; 3 PLK (Agnelli et al. 2009) [66]

1 (Zhan Myelona): 74 MM; 5 MGUS (Zhan et al. 2002) [67]

Additional files 2 and 3: Tables S2 and S3 summarize these data for MM and Pre-MM, respectively, with consideration of less stringent significance values. These alternative thresholds allowed for the classification of significance for several gene products that were very close to, but not quite at the point of significance, for the thresholds assumed by the publications. The gene products that were, thus, on the fringe, were considered. In one comparison, in which the borderline significant data were considered (data which were very close to the prescribed significance in the respective publications), the significance was considered ≤ 0.05 for Dataset 1 and was ≤ 0.000135 for Dataset 2. The data were also analyzed with regard to the less stringent significance thresholds: a threshold of ≤ 0.05 was considered for both Dataset 1 and 2; lastly, the threshold was adjusted to be ≤ 0.05 for both datasets. In addition, the reports published on the various gene products selected for this study were reviewed and several figures were created to summarize this information. Below is a summary of the specific literature findings regarding each gene product in question, with the expression expected from published literature noted along with the expression found herein the bioinformatics analysis from the Oncomine database. A comprehensive study has been reported investigating the role of the NF-κB/Snail/YY1/RKIP circuitry in multiple myeloma and how each gene is correlated with the remaining genes in this axis by examining various myeloma-related Gene Expression Omnibus (GEO) datasets [73].

**Fig. 3:**
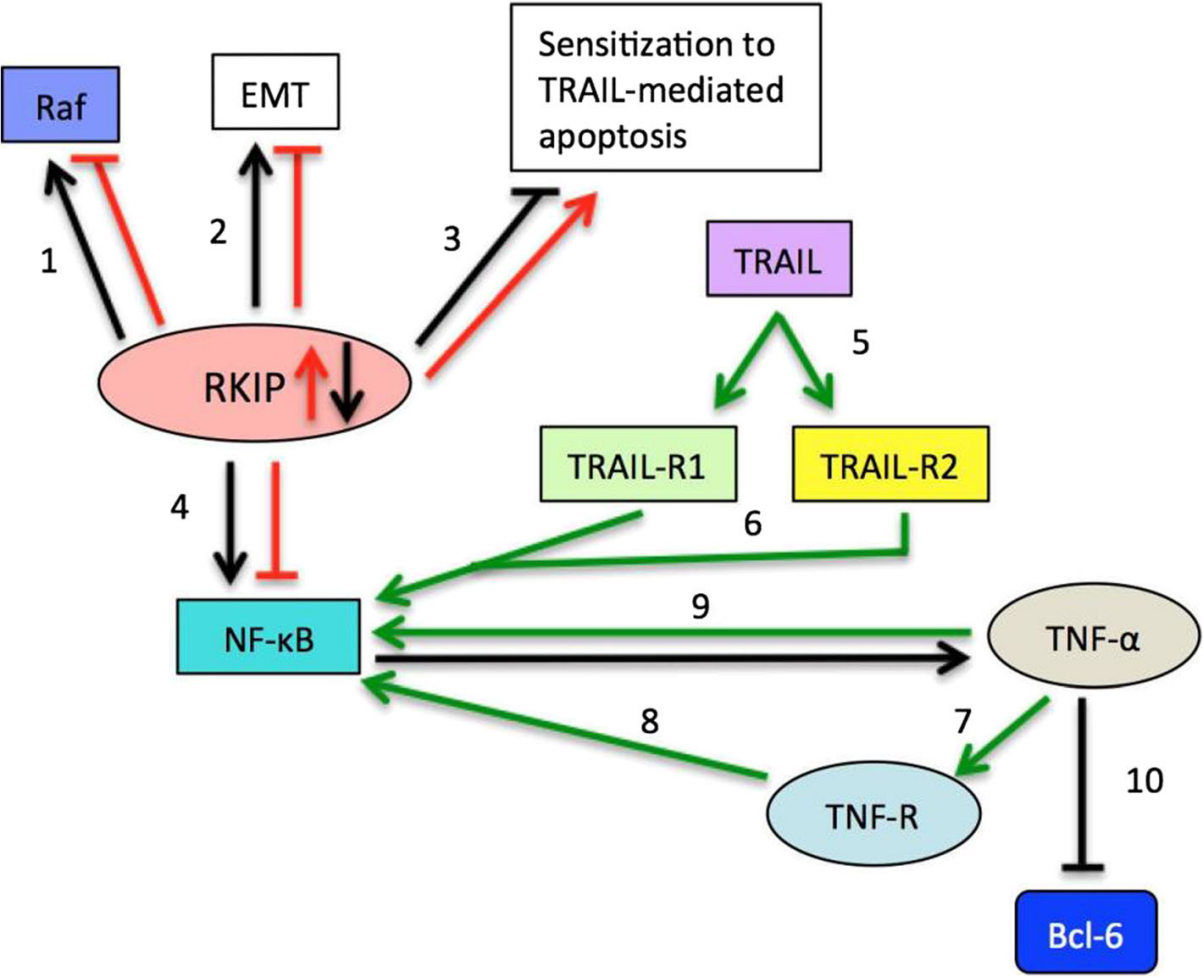
RKIP’s Downstream Targets. *Red arrows* depict the interactions of downstream gene products when RKIP expression is high and* black arrows* depict the interactions of downstream gene products when RKIP expression is low. 1: High expression of RKIP inhibits Raf, while low RKIP expression leads to Raf activation [15]; 2: Low RKIP is correlated with the epithelial to mesenchymal (EMT) transition [15]. Overexpression of Snail can inhibit RKIP and further induce EMT [81]; 3: RKIP overexpression sensitizes cells to TRAIL-mediated apoptosis [93]; 4: High expression of RKIP leads to inhibition of NF-κB, while low RKIP expression leads to high expression of NF-κB [15]; 5: TRAIL binds to its receptors, TRAIL-R1 and TRAIL-R2 [94]; 6: Upon binding to TRAIL-R1 or TRAIL-R2, TRAIL has been shown to stimulate activation of the NF-κB [94]; 7: TNF-α can stimulate TNFR-positive tumor cells [95]. TNF-α binds to its receptors, TNFR-1 and TNFR-2 [95]; 8: Upon stimulation of TNFR-positive tumor cells by TNF-α, cells can undergo activation of NF-κB [95]; 9: TNF-α can induce constitutive activation of NF-κB [63 via NF-kappaB and YY1’, in the regulation of tumor cell resistance to Fas-induced apoptosis]; 10: NF-κB mediates TNF-α- induced Bcl-6 expression [62]

As noted previously, Additional file 4: Figure S1 gives a broad overview of the various interactions of RKIP and its network of downstream effectors. Figure 3 summarizes the literature findings of the immediate downstream targets of RKIP, which are Raf and NF-κB, in addition to the interaction of TRAIL and its associated downstream targets [15, 93].

**Fig. 4:**
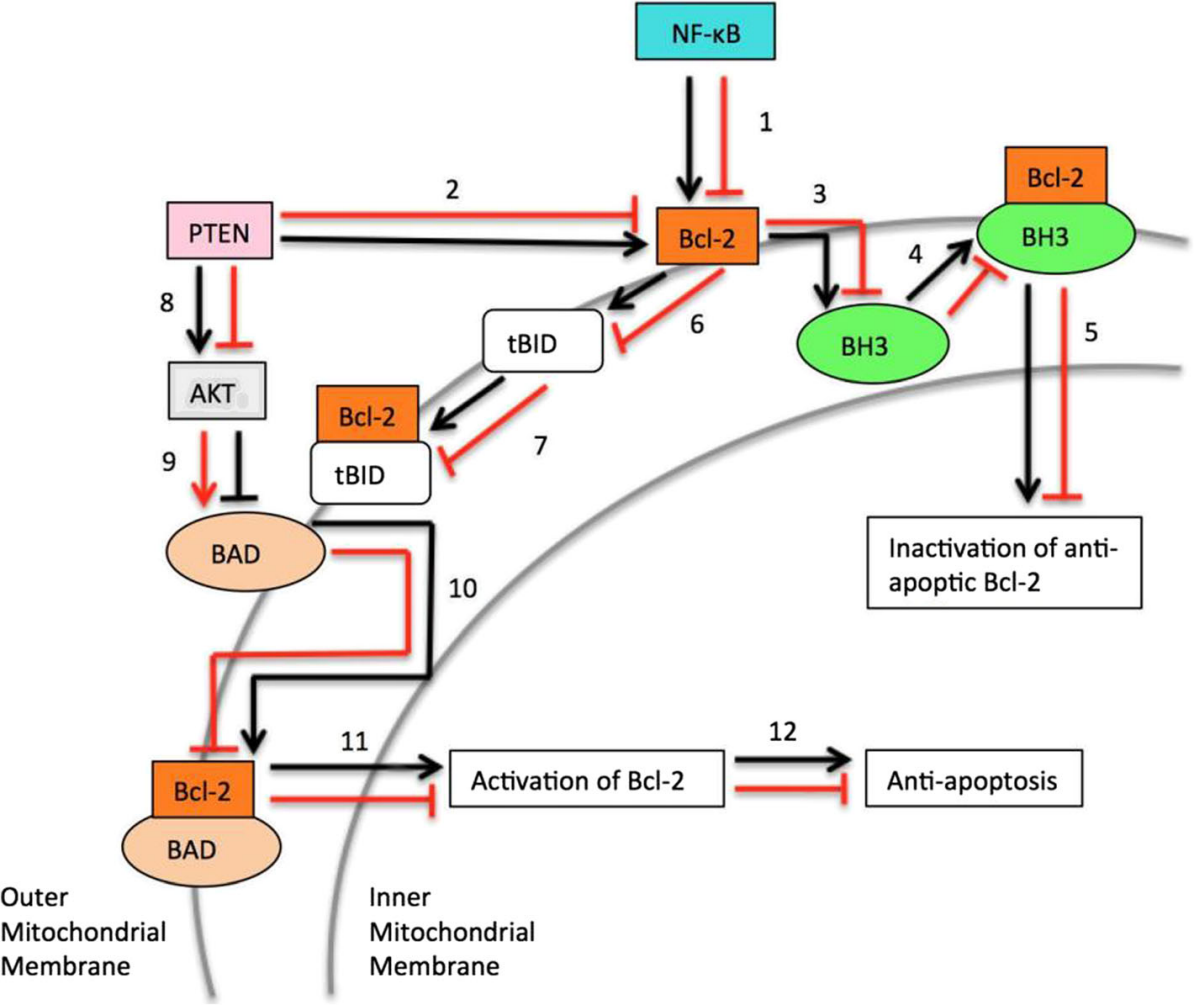
Bcl-2 Regulation. *Red arrows* depict the interactions of downstream gene products when RKIP expression is high and *black arrows* depict the interactions of downstream gene products when RKIP expression is low. 1: Under normal conditions, high RKIP leads to low NF-κB expression, and thus Bcl-2 expression is low. In cancer cells, low RKIP expresses leads to high NF-κB and thus high Bcl-2 expression [61]; 2: Pten expression inhibits Bcl-2 [9]; 3: When Bcl-2 is expressed, it may alternatively bind BH3 [96, 97]; 4: Available Bcl-2 can form a bound complex with BH3 [96, 97]; 5: When Bcl-2 is bound to BH3, this leads to inactivation of Bcl-2, which has anti-apoptotic activity. Thus, if Bcl-2 is inactivated, apoptosis can occur [96, 97]; 6: Bcl-2 sequesters tBID (truncated BID) [96, 97]; 7: Bcl-2 can form a complex with tBID [96, 97]; 8: When RKIP expression is high normally, this leads to downstream activation of Pten, which inhibits AKT. When RKIP expression is low, this leads to low Pten levels, and thus AKT is expressed [13]; 9: AKT expression leads to BAD inactivation, whereas low AKT allows for available activated BAD [96, 97]; 10: When BAD is in its activated form, in can react with Bcl-2. Calcineurin B complex activates BAD, which replaces tBID, thus binding Bcl-2 [96, 97]; 11: When BAD binds Bcl-2, this leads to Bcl-2 activation [96, 97]; 12: The activation of Bcl-2 leads to anti-apoptotic activity. When Bcl-2 is not activated, apoptosis can occur [96, 97]

Figure 4 depicts the regulation of Bcl-2 by NF-κB and PTEN, as well as the process of activation and inactivation of Bcl-2 [61, 69].

The data mined from Oncomine report the differences in the expression of gene products between Pre-MM and MM samples. Many gene products had no significant variations, especially those in the pre-MM condition, which may be due to the small pre-MM sample size. Several gene products did have different expressions in MM and pre-MM, which may be significant in that these gene products could play as important factors in the MM progression. However, a few gene transcripts had modified expressions in MM as compared to pre-MM. A summary of the gene products whose expressions were determined via the bioinformatics data to be significantly overexpressed or underexpressed with accordance to published significance values in MM and Pre-MM is depicted in Table 3. Gene products’ expressions were also summarized according to the less stringent alternative thresholds (shown in Additional files 1 and 2: Tables S1 and S2) in Additional file 5: Table S4.

**Table 3:** Summary of Gene product expression in MM and Pre- MM

Overexpression		Underexpression
Multiple Myeloma (MM)		
RKIP	DR5	Bcl-6
Bcl-2	PTEN	Fas
TRAIL	TNF-α	TNFR-2
Pre- Multiple Myelona (Pre- MM)		
Bcl-2	FasL	YY1
Bcl-6	TNF-α	

First, Bcl-6 was found to be overexpressed in Dataset 1 in Pre-MM, but was found to be underexpressed in Dataset 1 in MM. Bcl-2 was found to be overexpressed in Dataset 1 in Pre-MM and MM, but was found to be overexpressed in Dataset 2 in MM, indicating variability between sample conclusions among datasets. Lastly, TNF-α was found to be overexpressed in Dataset 1 in Pre-MM as well as in MM. Bcl-6 is upregulated in the bone marrow microenvironment in MM cells, while NF-κB mediates TNF-α- induced Bcl-6 expression. Thus, Bcl-6 expression is modulated, at least in part, via the Janus kinase/STAT3 and NF-κB pathways [62].

For certain gene products, a difference in their expression was noted amongst Pre-MM and MM cases. For instance, Fas was underexpressed in MM but was overexpressed in pre-MM. Bcl-6 was underexpressed in MM but was found to be overexpressed in dataset 1 and underexpressed in dataset 2 for the pre-MM condition. Similarly, in MM, TNF-α was overexpressed in dataset 1 but underexpressed in dataset 2, while in pre-MM, TNF-α was overexpressed. Lastly, in MM, YY1 was overexpressed, however, in pre-MM YY1 was underexpressed. These differences may suggest a correlation between MM progression and gene product expression in the case of YY1, Fas, Bcl-6, and TNF-α. Based on Fig. 2b, if RKIP expression is low, or in the case of MM, if there are high levels of inactive phosphorylated-RKIP, YY1 levels would be expected to rise and Fas levels would be expected to fall, which is only seen in the MM condition.

With regard to RKIP expression, the Oncomine analyses of the mRNA transcripts in MM did not discriminate between the two subsets of cells that may express RKIP or phospho-RKIP, which led to the creation of two possible mechanisms of action of RKIP and its downstream effectors in either the active non-phosphorylated or the inactive, phosphorylated case. It may be possible that phosphorylated RKIP could have some effect on some downstream effectors and not on others.

Since RKIP expression regulates both the NF-κB and MAPK survival pathways, the overexpression of “inactive” p-Ser153 RKIP in MM might contribute positively to the overall cell survival/antiapoptotic phenotype and drug resistance of MM through the constitutive activation of survival pathways and the downstream transcription of anti-apoptotic gene products, which can contribute to the overall cell survival and drug resistance [11, 60]

After analyzing these gene products on Oncomine, a schematic diagram was developed to attempt to explain different scenarios for RKIP functioning in MM and its downstream effectors. Since the RKIP from the samples was not distinguished from its phosphorylated or non-phosphorylated form, Figs. 2a, b were created to explore the possibilities that may ensue from either situation. Figure 2a summarizes the relationships between high functional RKIP expression and its corresponding pathways. Figure 2b summarizes the relationship as well as a correlation between inactive, phosphorylated RKIP, which is found in high levels in MM, and its potential effects on its downstream targets. The reported findings in the literature support some of the Oncomine bioinformatics expression findings (see above). Several of the gene products analyzed by Oncomine have expressions that provide support for Fig. 2a while other gene product expressions provide support for Fig. 2b. The overexpression of RKIP, DR5, and PTEN and the underexpression of Bcl-6 in the MM bioinformatics data support the proposed schematic in Fig. 2a. However, the overexpression of Bcl-2 and TNF-α and the underexpression of Fas in the MM bioinformatics data support the proposed schematic in Fig. 2b.

### Future Directions

For prognostic significance, it would be informative to identify the MM and pre-MM cells for their expression levels of RKIP and phosphorylated RKIP. Examining the mRNA transcripts from both MM and pre-MM following separation of the cells expressing RKIP or phosphorylated RKIP could help elucidate which downstream targets are being acted upon or not in each situation. If these findings are validated, they may provide additional therapeutic targets for treatment of MM.

## Additional files

Additional file 1: Table S1. Summary of Gene Product Overexpression/Underexpression in MM & Pre MM. (ZIP 294 kb)

Additional file 2: Table S2. Compilation of Bioinformatics MM Gene Product Expression Data with Various Significance Value Thresholds. (JPG 158 kb)

Additional file 3: Table S3. Compilation of Bioinformatics Pre- MM Gene Product Expression Data with various significance value thresholds. (JPG 222 kb)

Additional file 4: Figure S1. The RKIP/NF-κB/Snail/YY1/PTEN loop and its Downstream Targets. Red arrows depict the interactions of downstream gene products when RKIP expression is high and black arrows depict the interactions of downstream gene products when RKIP expression is low. 1: High expression of RKIP leads to inhibition of NF-κB, while low RKIP expression leads to high expression of NF-κB [15]; 2: Low levels of NF-κB leads to low CIAP1 and XiAP expression, while high NF-κB allows for their expression [61]; 3: When CIAP1 and XiAP are expressed, they inhibit apoptosis, but when their levels are low, apoptosis can occur [61]; 4: Expression of NF-κB leads to low levels of DR5 [13]; 5: Low NF-κB expression leads to low expression of YY1 [14]; 6: Low NF-κB expression leads to low expression of Snail [14]; 7: Low NF-κB leads to low Bcl-2 expression [61]; 8: Snail represses RKIP normally. Thus, when Snail expression is low, it allows for RKIP expression [14]; 9: Low levels of Snail correlate with high expression of Pten [13]; 10: Low levels of Snail allow for expression of E-cadherin [61]; 11: YY1 is a regulator of Snail, thus low YY1 expression leads to low Snail expression [13]; 12: Low levels of YY1 correlate with high expression of Pten [13]; 13: Low levels of YY1 correlate with the induction of Fas [13]; 14: Low levels of YY1 correlate with the induction of DR5 [13]; 15: Pten suppresses YY1 via its induction of HIF-2-α transcriptional activity [13]; 16: Pten expression inhibits Bcl-2 [9]; 17: Pten expression inhibits AKT [13]; 18: AKT phosphorylates XiAP, preventing it from being degraded. Thus, low AKT levels would allow for XiAP degradation and thus lead to low XiAP levels [98]; 19: AKT expression causes inactivation of BAD via phosphorylation. Thus, low AKT expression does not lead to BAD phosphorylation and thus is associated with free BAD [96, 97]; 20: AKT expression leads to BAD inactivation, whereas low AKT allows for available activated BAD [96, 97]. (JPG 113 kb)

Additional file 5: Table S4. Summary of Gene Product expression in MM and Pre- MM considering Least Stringent Alternative threshold. (JPG 113 kb)

## Abbreviations

MM: Multiple myeloma
pRKIp: Phosphorylated Raf kinase inhibitor protein
PTEN: Phosphatase and tensin homolog
RKIP: Raf kinase inhibitor protein
Snai1: Snail Family Zinc Finger 1
Snai2: Snail Family Zinc Finger 2
YY1: Yin Yang 1
