# Supplementary figures and images for: Overexpression of RKIP and its cross-talk with several regulatory gene products in multiple myeloma
Source: J Exp Clin Cancer Res. 2017 May 5;36:62. doi: 10.1186/s13046-017-0535-z (PMC5420138; doi:10.1186/s13046-017-0535-z)

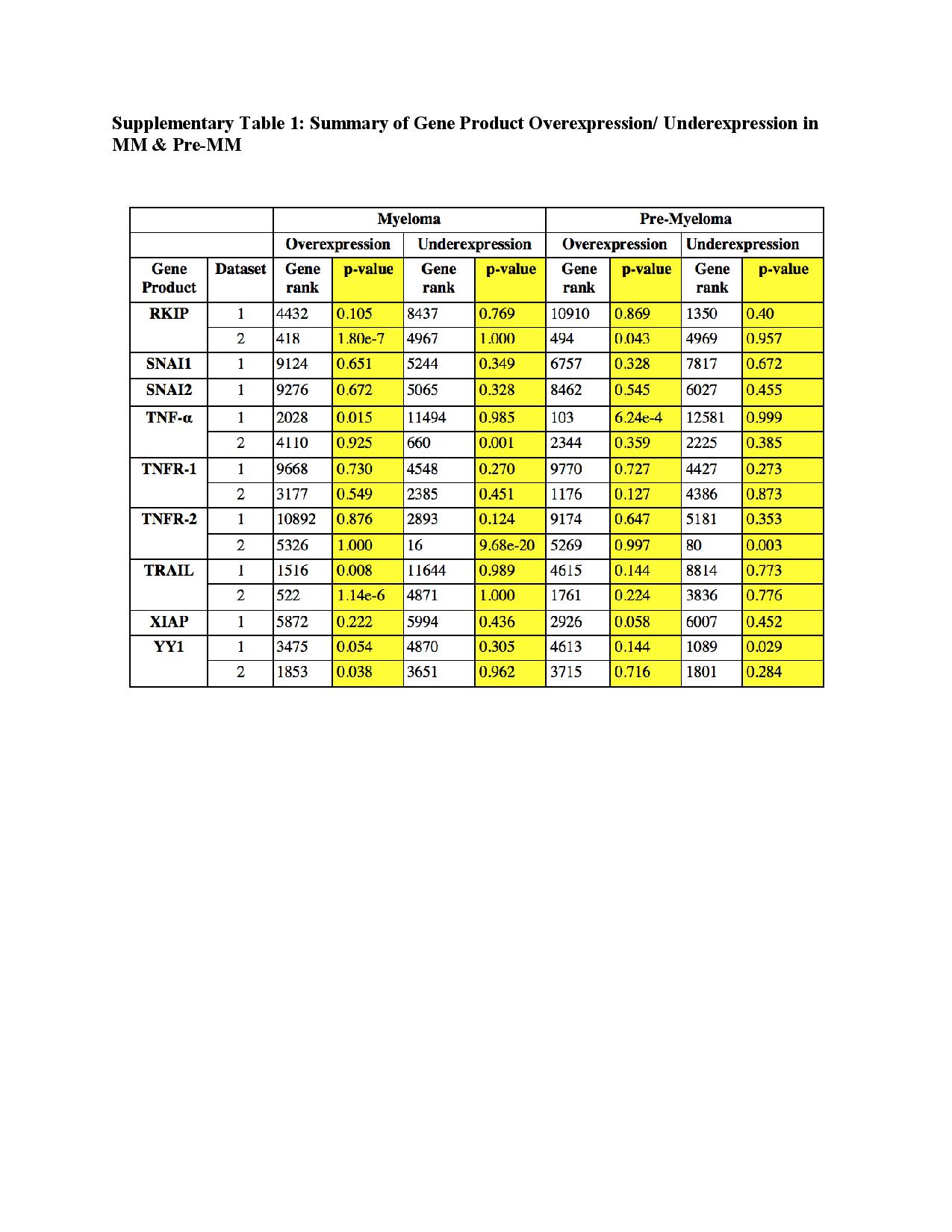

Supplement: Supplementary file 1 — Summary of Gene Product Overexpression/Underexpression in MM & Pre MM. (ZIP 294 kb) [file 13046_2017_535_MOESM1_ESM.zip › Supplementary Table 1.jpg]

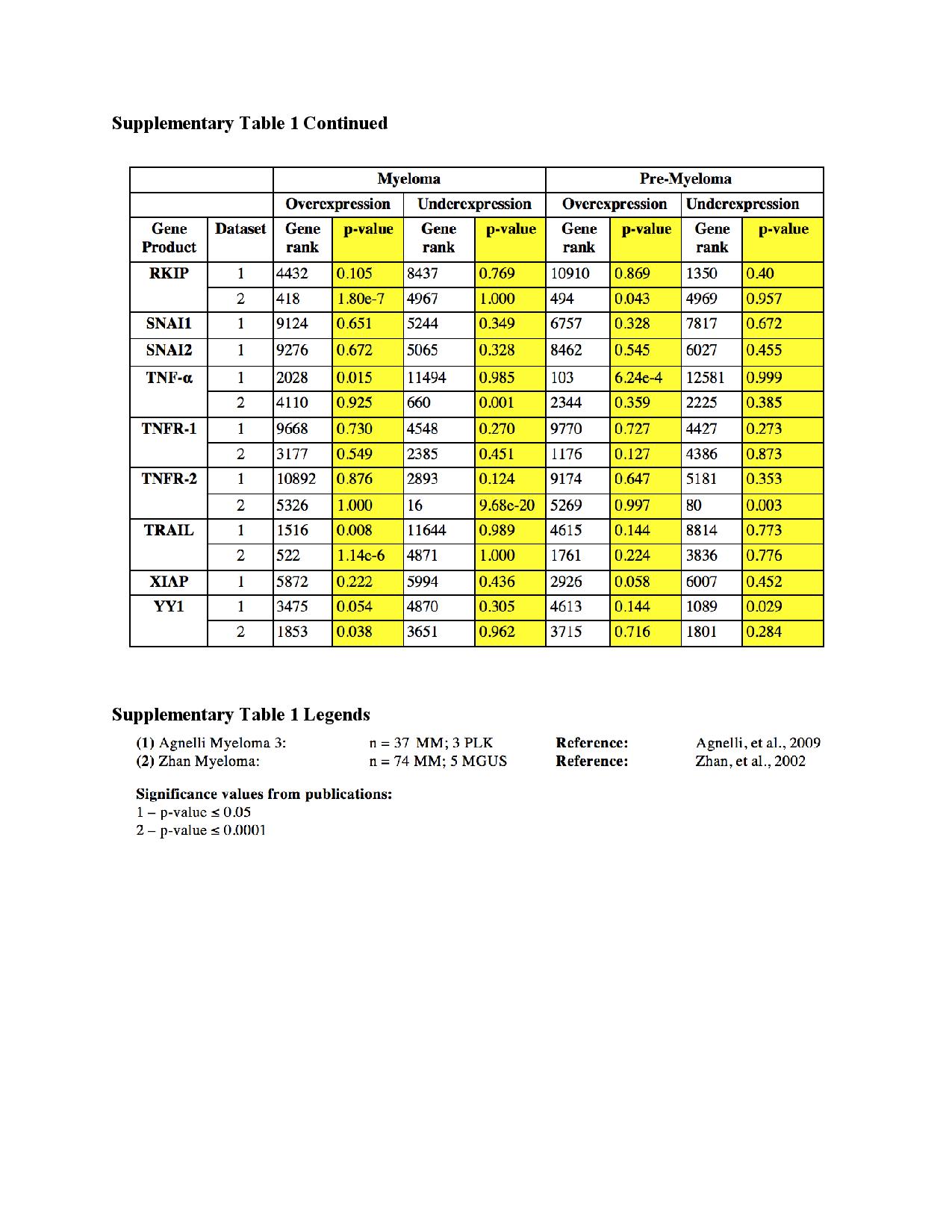

Supplement: Supplementary file 1 — Summary of Gene Product Overexpression/Underexpression in MM & Pre MM. (ZIP 294 kb) [file 13046_2017_535_MOESM1_ESM.zip › Supplementary Table 1 Continued.jpg]

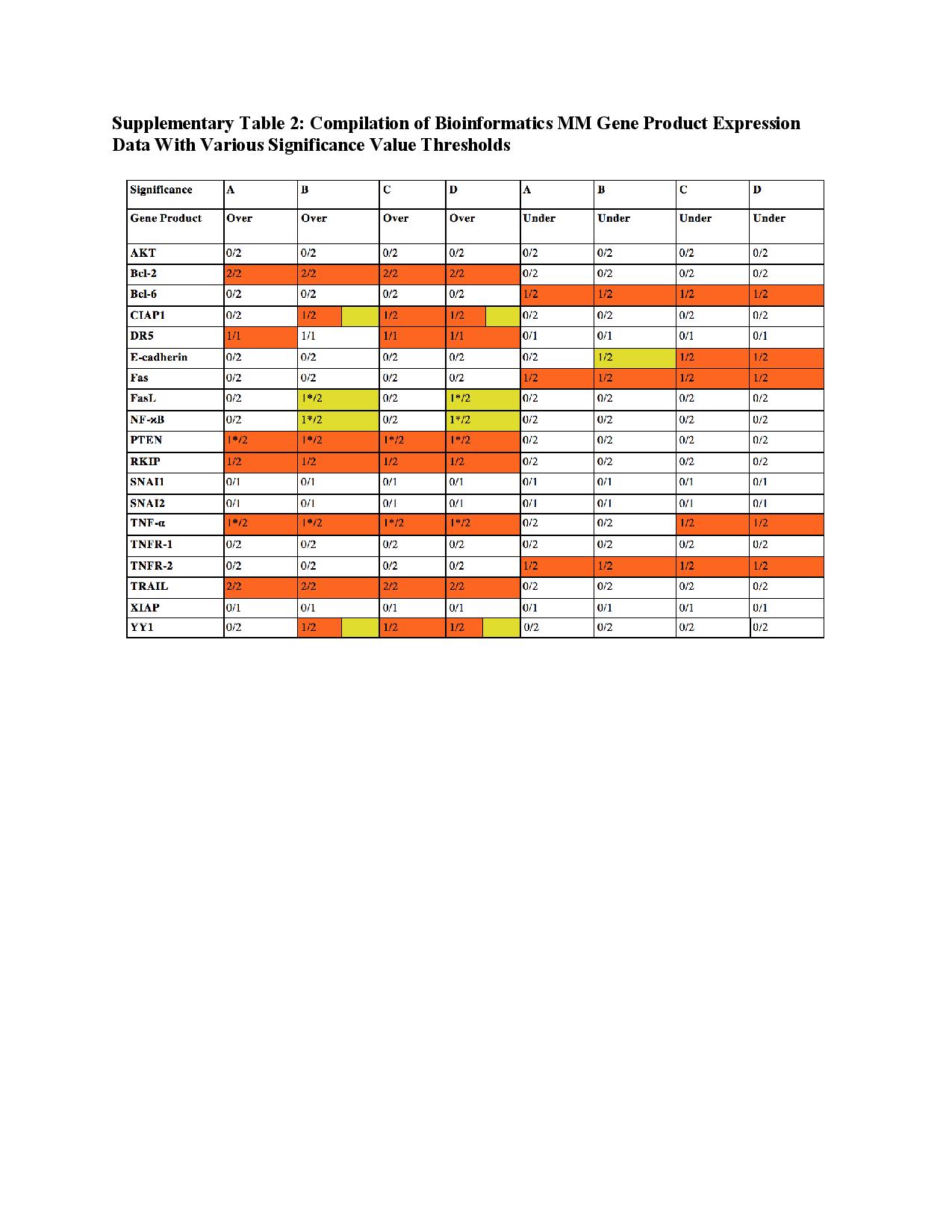

Supplement: Supplementary file 2 — Compilation of Bioinformatics MM Gene Product Expression Data with Various Significance Value Thresholds. (JPG 158 kb) [file 13046_2017_535_MOESM2_ESM.jpg]

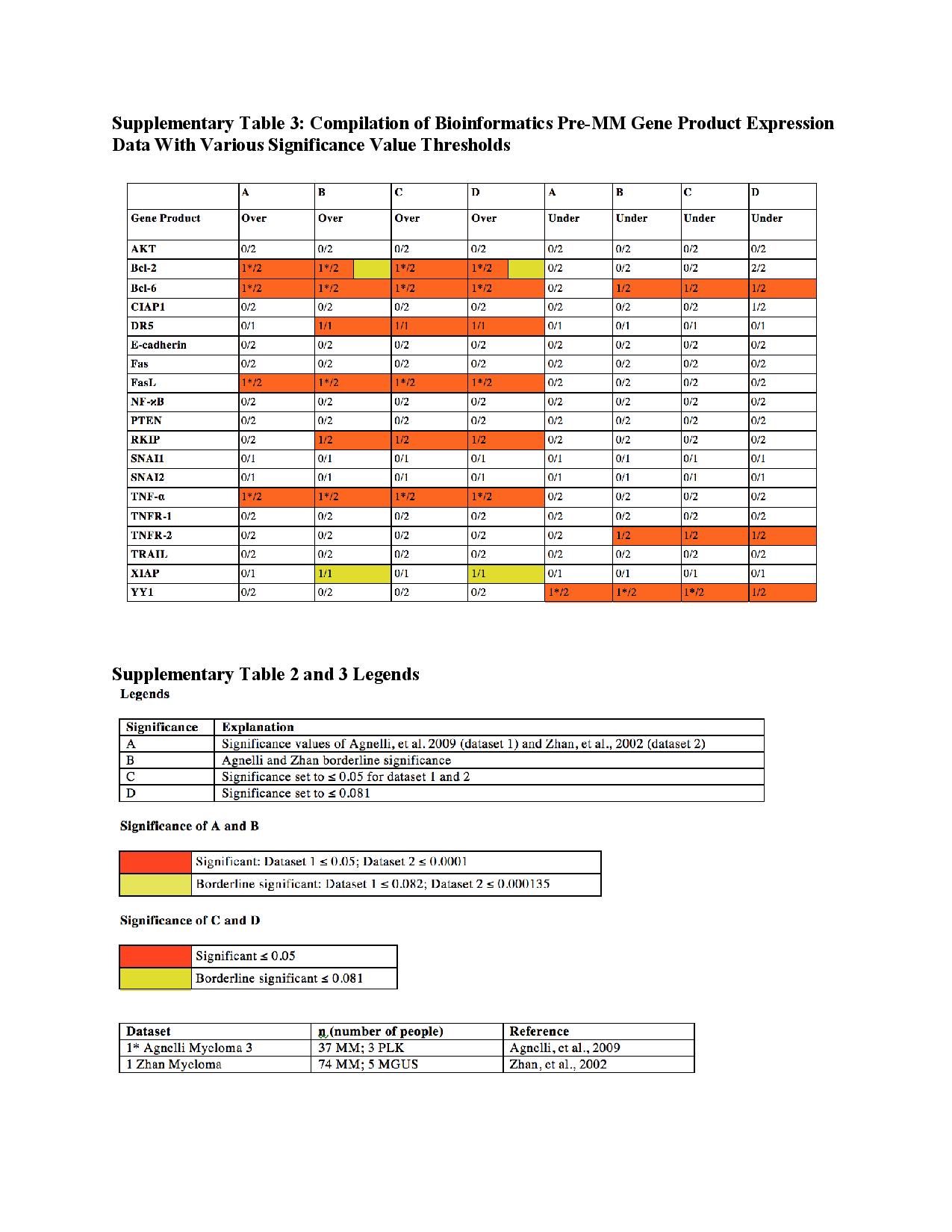

Supplement: Supplementary file 3 — Compilation of Bioinformatics Pre- MM Gene Product Expression Data with various significance value thresholds. (JPG 222 kb) [file 13046_2017_535_MOESM3_ESM.jpg]

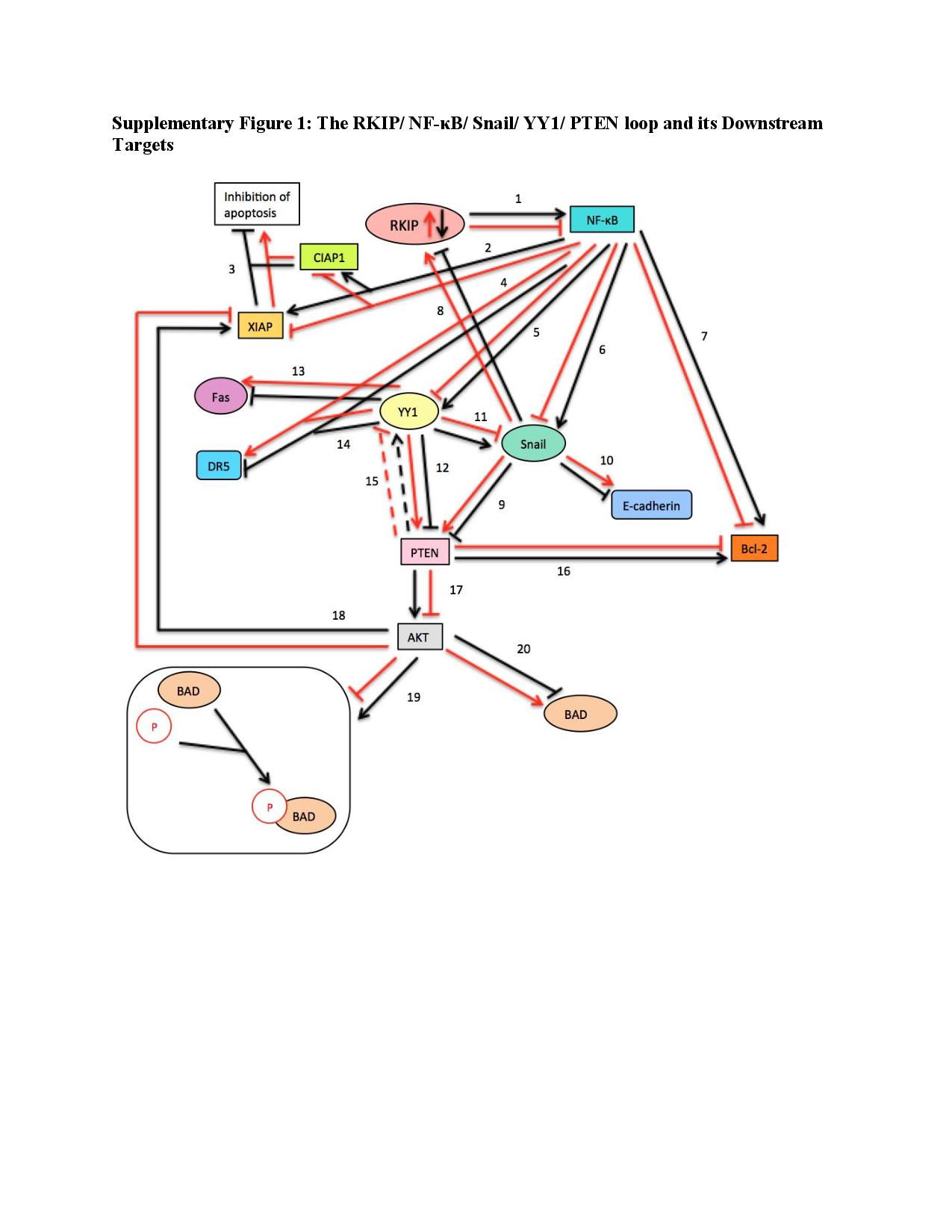

Supplement: Supplementary file 4 — The RKIP/NF-κB/Snail/YY1/PTEN loop and its Downstream Targets. Red arrows depict the interactions of downstream gene products when RKIP expression is high and black arrows depict the interactions of downstream gene products when RKIP expression is low. 1: High expression of RKIP leads to inhibition of NF-κB, while low RKIP expression leads to high expression of NF-κB [15]; 2: Low levels of NF-κB leads to low CIAP1 and XiAP expression, while high NF-κB allows for their expression [61]; 3: When CIAP1 and XiAP are expressed, they inhibit apoptosis, but when their levels are low, apoptosis can occur [61]; 4: Expression of NF-κB leads to low levels of DR5 [13]; 5: Low NF-κB expression leads to low expression of YY1 [14]; 6: Low NF-κB expression leads to low expression of Snail [14]; 7: Low NF-κB leads to low Bcl-2 expression [61]; 8: Snail represses RKIP normally. Thus, when Snail expression is low, it allows for RKIP expression [14]; 9: Low levels of Snail correlate with high expression of Pten [13]; 10: Low levels of Snail allow for expression of E-cadherin [61]; 11: YY1 is a regulator of Snail, thus low YY1 expression leads to low Snail expression [13]; 12: Low levels of YY1 correlate with high expression of Pten [13]; 13: Low levels of YY1 correlate with the induction of Fas [13]; 14: Low levels of YY1 correlate with the induction of DR5 [13]; 15: Pten suppresses YY1 via its induction of HIF-2-α transcriptional activity [13]; 16: Pten expression inhibits Bcl-2 [9]; 17: Pten expression inhibits AKT [13]; 18: AKT phosphorylates XiAP, preventing it from being degraded. Thus, low AKT levels would allow for XiAP degradation and thus lead to low XiAP levels [98]; 19: AKT expression causes inactivation of BAD via phosphorylation. Thus, low AKT expression does not lead to BAD phosphorylation and thus is associated with free BAD [96, 97]; 20: AKT expression leads to BAD inactivation, whereas low AKT allows for available activated BAD [96, 97]. (JPG 113 kb) [file 13046_2017_535_MOESM4_ESM.jpg]

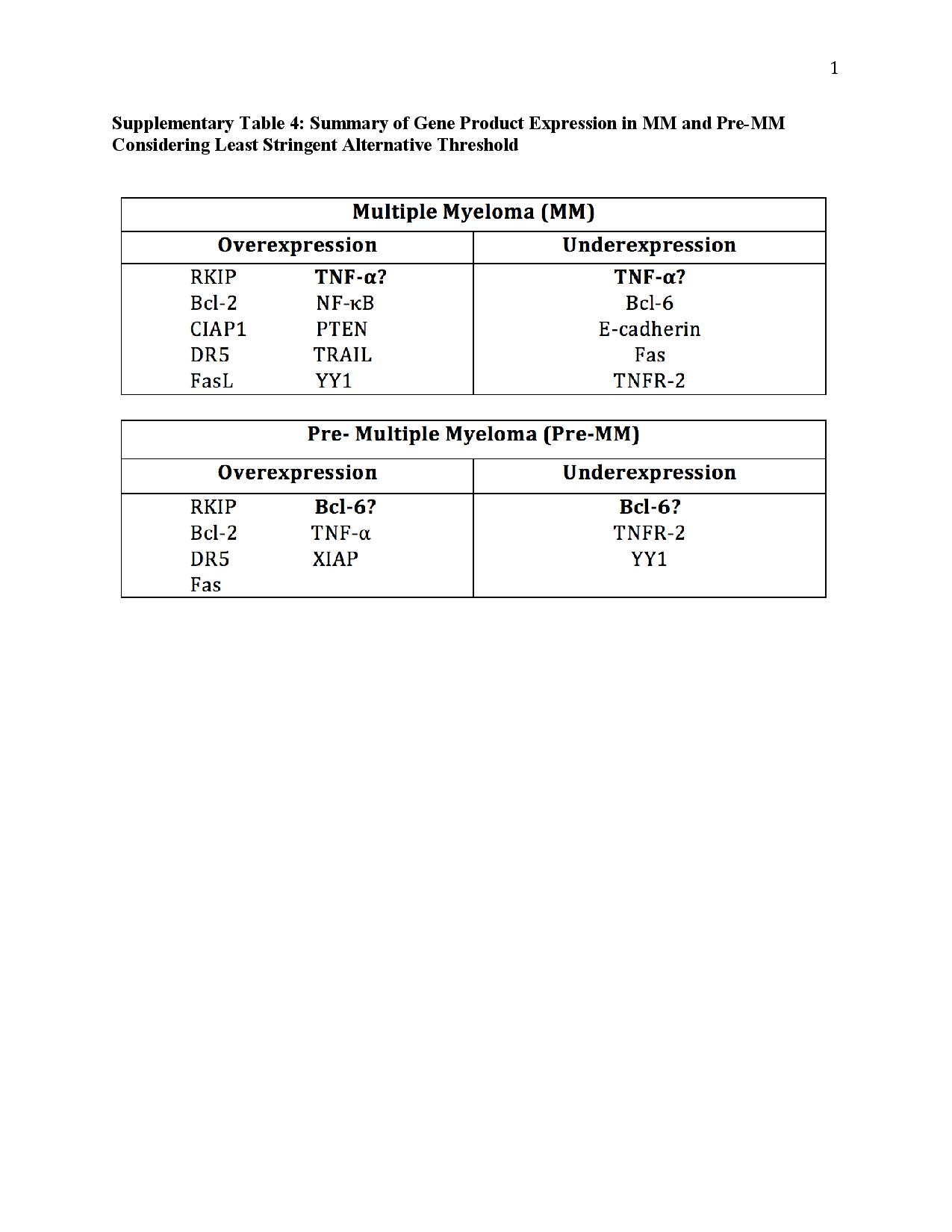

Supplement: Supplementary file 5 — Summary of Gene Product expression in MM and Pre- MM considering Least Stringent Alternative threshold. (JPG 113 kb) [file 13046_2017_535_MOESM5_ESM.jpg]
